# Promotion of angiogenesis by M13 phage and RGD peptide in vitro and in vivo

**DOI:** 10.1038/s41598-019-47413-z

**Published:** 2019-08-01

**Authors:** Zohreh Safari, Sara Soudi, Nazli Jafarzadeh, Ahmad Zavaran Hosseini, Elham Vojoudi, Majid Sadeghizadeh

**Affiliations:** 1 Department of genetics, Faculty of Biological Sciences, Tarbiat Modares University, Tehran, Iran; 2 Department of Immunology, Faculty of Medical Sciences, Tarbiat Modares University, Tehran, Iran; 3 Department of Regenerative Medicine, School of Advanced Technologies in Medicine, Tehran University of Medical Sciences, Tehran, Iran

**Keywords:** Gene expression, Cell migration, Angiogenesis

## Abstract

One of the most important goals of regenerative medicines is to generate alternative tissues with a developed vascular network. Endothelial cells are the most important cell type required in angiogenesis process, contributing to the blood vessels formation. The stimulation of endothelial cells to initiate angiogenesis requires appropriate extrinsic signals. The aim of this study was to evaluate the effects of M13 phage along with RGD peptide motif on *in vitro* and *in vivo* vascularization. The obtained results demonstrated the increased cellular proliferation, HUVECs migration, cells altered morphology, and cells attachment to M13 phage-RGD coated surface. In addition, the expression of Vascular Endothelial Growth Factor A (VEGF-A), VEGF Receptors 2 and 3, Matrix Metalloproteinase 9 (MMP9), and epithelial nitric oxide synthase (eNOS) transcripts were significantly upregulated due to the HUVECs culturing on M13 phage-RGD coated surface. Furthermore, VEGF protein secretion, nitric oxide, and reactive oxygen species (ROS) production were significantly increased in cells cultured on M13 phage-RGD coated surface.

## Introduction

Generating new tissues that can be used as alternative organs for damaged tissues is one of the important aims in tissue engineering^1^. Successful tissue engineering critically depends on developing a system for the distribution of oxygen and nutrients to all cells within a tissue via a vascular network^2,3^. There have been significant advances in the angiogenesis process. Endothelial cells (ECs) are considered as the most important cell source required for the angiogenesis process, forming the inner cell layer for blood vessels. The stimulation of ECs to initiate angiogenesis requires to an increase in the level of pro-angiogenic growth factors and appropriate extracellular matrix which subsequently plays an important role in this process^4,5^.

A large body of research conducted on tissue engineering aimed to design strategies for improving angiogenic potential of engineered tissues using bioscaffolds. It should be noted that the recent advances in tissue engineering field have been led to the design of biomaterials with proper physical characteristics of vascular tissue microenvironment. In addition, a suitable biomaterial used for this purpose should have the ability to induce proliferation and extracellular matrix component secretion by endothelial cells^6^.

Previous studies indicated that indirect factors in an engineered tissue could stimulate the resident cells to create an angiogenic microenvironment. The advantage of this method is in stimulating *in vivo* responses of cells in order to regulate factor secretion based on the tissues requirement during the angiogenesis^7^.

RGD (Arginine-glycine-aspartic acid) is a peptide cell adhesion motif displayed on various proteins of plasma and extracellular matrix (ECM). RGD plays important roles in the regulation of many biological activities such as cell attachment and spreading and the formation of cytoskeleton and focal adhesion, involved in signal transduction for modulating cellular behavior^8^. Gelatin and RGD are among the main biomaterials used for angiogenesis induction^7^. Previous reports demonstrated that the use of gelatin structures in bioscaffolds enhances the proliferation of endothelial and smooth muscle cells^9^.

In the previous study, M13 phage-based gene delivery methods were used for therapeutic purposes^10^. Interestingly, recent studies have demonstrated that M13 phage structures have the potential to be used as bioengineering scaffolds for stimulating regenerative responses *in vitro*^11–13^. It is worth noting that the reproduction of M13 phage in large scale is possible by simple bacterial amplification. The resulting phages are as the promising candidates for the construction of two- and three-dimensional scaffolds^14^. Hence, M13 phages with the ability of fixing biochemical ligands may be useful for future studies conducted on scaffolding and regenerative medicine.

Therefore, the aim of the current study was to evaluate the role of M13 phage and RGD peptide motif in stimulating angiogenic responses of *in vitro* cultured endothelial cells and *in vivo* implanted PLGA/PCL scaffolds. In particular, cell viability and migration, the expression of angiogenic genes, and the secretion of angiogenic factors including VEGF and nitric oxide were assessed on selected surfaces, including coated with M13 phage-RGD, M13 phage, and gelatin and unmodified control surfaces after 48 hrs of HUVECs culturing. Moreover, *in vivo* gene expression analysis showed significantly enhanced expression of angiogenic factors in scaffolds of M13 phage-RGD group. Accordingly, histological analysis of implanted scaffolds exhibited increased vascularization potential in sectioned tissues of M13 phage-RGD group compared to other experimental groups.

## Materials and Methods

### Large-scale amplification of M13 bacteriophage

M13 bacteriophages were amplified and purified as described previously with some modifications^15,16^. Briefly, 500 mL of *Escherichia coli* TG1 culture was added to 2YTX medium, and bacteria were grown to mid-log phase and then infected with 1 mL of wild-type M13 phage (10^12^ PFU.mL^−1^). The cultures were incubated at 37 °C for 6 hours (shaking is required) and subsequently centrifuged for 30 minutes at 8000 g to remove bacterial cells. Finally, bacteriophages were collected by subsequent centrifuging at 20000 g for 150 minutes. The pellet of previous step was re-suspended in phosphate buffered saline (PBS) (Gibco, USA), and the concentration of isolated bacteriophage was determined spectrophotometrically using an extinction coefficient of 3.84 cm^2^.mg^−1^ at 269 nm.

### Transmission electron microscopy (TEM)

For TEM analysis, 20 µL of M13 bacteriophage solution was exposed to a 300-mesh carbon-coated copper grid (AGS160-3), and the grid was negatively stained with 2% uranyl acetate. After staining, the grid was rinsed with sterile distilled water. Images were taken up by a Zeiss - EM10C transmission electron microscope (Germany) at a voltage of 80 KV.

### Cell culture

HUVECs cell line was purchased from Pasteur institute and cultured in DMEM (Gibco, USA) supplemented with 10% fetal bovine serum (FBS) (Gibco, USA) and 1% penicillin/streptomycin.

### Cell viability assay

Biocompatibility of M13 bacteriophage (at a concentration of 10^12^ pfu. mL^−1^) was examined by MTT (3-[4,5-dimethylthiazol-2-yl]-2,5-diphenyltetrazolium bromide) (Sigma-Aldrich, USA) assay. Briefly, HUVECs were cultured on surfaces coated with M13 phage, M13 phage-RGD, and gelatin and control plates (no coated surface) at a density of 10^4^ cells per well of 96 well. After 48 hours, MTT solution (20 μL of 5 mg. mL^−1^ MTT solution) was added to each well, and plates were incubated at 37 °C for 3–4 hours. Then, the medium was discarded, and formazan crystals were dissolved by adding 200 μL of dimethyl sulfoxide (Sigma-Aldrich, USA). The optical density was measured at 570 nm using a microplate scanning spectrophotometer (BioTek, USA).

### Cell apoptosis analysis

The effects of coated surfaces on HUVECs apoptosis were determined using Annexin V-FITC/PI apoptosis detection kit (Roche, Germany). Briefly, cells were seeded on selected surfaces and incubated for 48 hours in CO_2_ incubator. Then, cells were trypsinized, washed with PBS, and stained with binding buffer containing Propidium iodide (PI) and FITC-conjugated anti-Annexin V antibody. The rate of apoptosis was determined using a FACSCalibur cytometer (USA).

### Scanning electron microscopy (SEM)

In the next step, the morphology of HUVECs was identified using SEM in order to observe cells detailed morphological changes in experimental groups. First, the cells were cultured on pre-coated slides with gelatin, M13 phage, and M13 phage-RGD and control unmodified slide. After 48 hours, the slides were air-dried, and then the slides were gold coated. The samples were imaged using a scanning electron microscope (KYKY-EM3200, 26 KV).

### Gelatin zymography

For the analysis of MMP9 activity in the supernatant of HUVECs of each group, equal volumes of supernatant (20 µL) were loaded on a SDS-polyacrylamide gel (10%) including 0.1% gelatin (Sigma-Aldrich, USA). After washing with 2.5% Triton-X 100 for 20 minutes (two times), gels were incubated in developing buffer (including NaCl, CaCl_2_, and Tris) for 16 hours at 37 C. Next, gels were stained with a 10% acetic acid and 25% methanol solution containing 0.5 mg. mL^−1^ Coomassie Brilliant Blue R-250 (Sigma-Aldrich, USA) and gently agitated for 2 hours. In addition, destination was performed by 8% acetic acid and 4% methanol for 2 hours. Finally, gels were photographed, and the images were analyzed using ImageJ software (version 1.8.0).

### Wound healing assay

HUVECs were seeded on 12-well tissue culture plates (wells pre-coated with M13 phage, M13 phage-RGD, and gelatin). After reaching confluency, a scratch wound was created on the cell monolayer with yellow pipette tips. After that, medium was carefully changed to remove debris, and cells were incubated at 37 °C in incubator (5% CO_2_). The scratched area of each experimental group was imaged with an inverted microscope (Olympus, Japan) at 0, 9, and 18 hours, respectively^17^.

### Cytokine secretion and nitric oxide production

Supernatants of the cells in each experimental group were collected, and the level of VEGF protein was determined using ELISA kits (Abcam, UK) according to the manufacturer’s protocol. Absorbance of each sample was quantified using a BioTek microplate reader (USA) at 450 nm.

Nitric oxide production was determined by measuring the accumulation of NO_2_ in the supernatants of HUVECs cultured on selected surfaces by Griess reaction, as previously described^18^. Briefly, equal volumes of Griess reagent and cell culture supernatants were mixed and incubated at room temperature for 10 minutes. After that, the absorbance of each sample was determined at 540 nm using a BioTek microplate reader (USA). Serial dilutions of NO_2_ was used to establish the standard curve.

### RNA preparation, cDNA synthesis, and qRT-PCR

In each experimental group, cells were subjected into RNA extraction after 48 hours culturing using RiboEX (GeneAll) according to the manufacturer’s instructions. Of the total RNAs extracted from each experimental group, 1 µg was treated with DNase I enzyme (Thermo, USA), and subsequently DNase treated RNA was subjected into reverse transcription (Thermo, USA). RT^2^ SYBR Green High ROX Master mix (Biofact, Korea) was used for qRT-PCR, and data were quantified using ∆∆CT method. The sequences of each primer are in Supplementary Table S1.

### Measurement of intracellular ROS generation

The generation of intracellular ROS in HUVECs cultured on each selected surface was measured by flow cytometry technique using peroxide-sensitive 2- and 7-dichlorodihydrofluorescein diacetate (DCFH-DA) (Sigma-Aldrich, USA). After entry into a cell, DCF-DA underwent deacetylation process, and subsequently, the presence of intracellular ROS led to the oxidization of this molecule to form highly fluorescent DCF^19^. After 48 hours, cells cultured on each selected surface were washed and incubated with 10 μM DCFH-DA for 45 minutes at 37 °C. Then cells were washed twice with PBS and analyzed by flow cytometry (BD FACSCanto II, BD Bioscience, San Diego, CA, USA). Data were analyzed using Flowing software version 2-1.

### Preparation and electrospinning of nanofibrous scaffolds

In this study, PLGA (Sigma-Aldrich, USA) polymer was used to enhance cell adhesion, and PCL (Sigma-Aldrich, USA) polymer was used to increase the strength as well as to reduce degradation rate of our scaffolds.

The scaffolds were designed in four groups including: 3 PLGA/PCL sheets containing M13 phage, M13 phage-RGD, gelatin and one PLGA/PCL sheet with no extra material as a control group. For the scaffolds preparation, PLGA was dissolved in Chloroform (Carlo Ebra, France) 90% and N′N-dimethylformamide (DMF) (Merck German) 10% at a concentration of 17% (wt/wt) and stirred for 1 hour at room temperature. The solution was aspirated by a 5 mL syringe with a needle gauge of 20, attached to a high voltage. M13 phage, M13 phage-RGD, and gelatin were used in the PLGA solution for the experimental groups.

To prepare the PCL spinning solution, a homogeneous aqueous solution (10% weight per volume, wt/vol) was made by dissolving PCL in acetic acid. The solution was stirred for 24 hours at room temperature. The clear solution was electrospun by a 5 mL syringe with a needle gauge of 22 and mass flow rate of 1 mL/hour.

Sheets were fabricated using a laboratory electrospinning machine consist two syringes and a receiver drum. (diameter = 150 mm; length = 500 mm). The distance between the tip of the syringes and the collector was 8 cm, and the rotation rate of the mandrel was approximately 1000 rpm.

A high voltage (the applied voltage can be adjusted between 15–20 kV) was applied to the tip of the needles attached to the syringes when a fluid jet was ejected.

### *In vivo* experiments and histological analysis

All animal experiments were performed according to the Tarbiat Modares university ethical committee. The methods were carried out in accordance with the approved guidelines.

For *in vivo* studies, electrospun PLGA/PCL scaffolds were sterilized using 70% ethanol and rinsed with deionized water. Then the scaffolds were exposed to UV light on each side for 60 minutes. PLGA/PCL scaffolds (in combination with gelatin, M13 phage, and M13 phage-RGD) were transplanted into the subcutaneous and peritoneal cavity of 6-week-old female Balb/c mice (one implantation per animal) (n = 3). At day 21, the mice were ethically sacrificed, and the matrices were recovered. All RNAs of each experimental group scaffolds were extracted using RiboEX total RNA reagents and analyzed to determine the expression level of VEGF-A, VEGFR-2, VEGFR-3, MMP9, and eNOS gene. Moreover, the scaffolds were fixed in 10% formalin (Merck, Germany) and embedded in paraffin for histological analysis. Paraffin-embedded scaffolds were sectioned, stained with H&E, and imaged using Olympus light microscope (Japan) at ×20 magnifications.

### Statistical analysis

All statistical analyses were performed using GraphPad Prism software (version 6), and data were presented as mean ± standard error of means. Significant differences were determined using Student’s T test and one-way analysis of variance (ANOVA), and $P\,{\rm{v}}{\rm{a}}{\rm{l}}{\rm{u}}{\rm{e}}\le 0.05$ was considered as statistically significant.

## Results

### Characterization of M13 phage nanostructures

**Figure 1:**
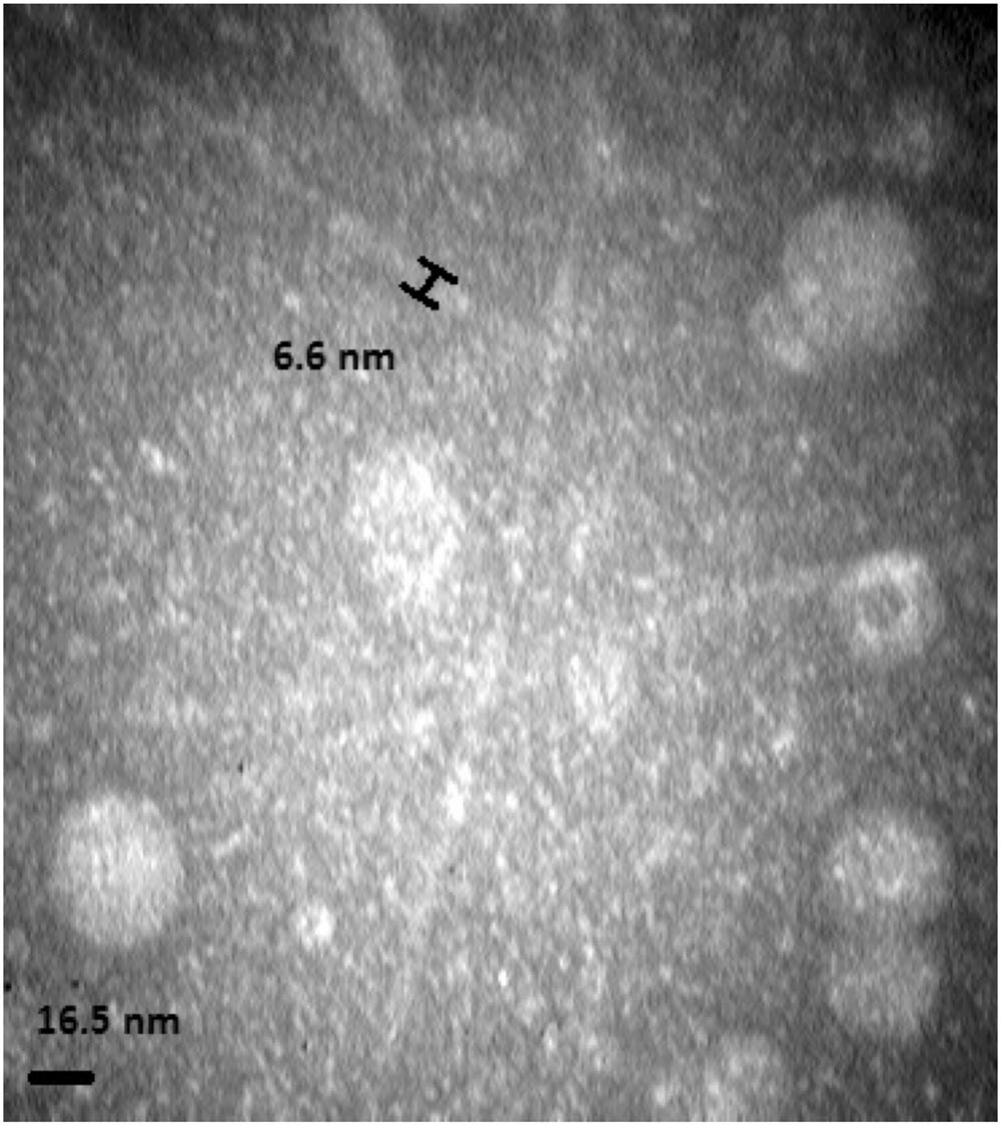
Transmission electron microscopic (TEM) view of M13 filamentous bacteriophages.

The results of TEM imaging showed that isolated phages represented the typical morphology of native M13 phage with a diameter of 6.6 nanometer (Fig. 1).

### The Effects of M13 phage, M13 phage with RGD, and gelatin on HUVECs viability

**Figure 2:**
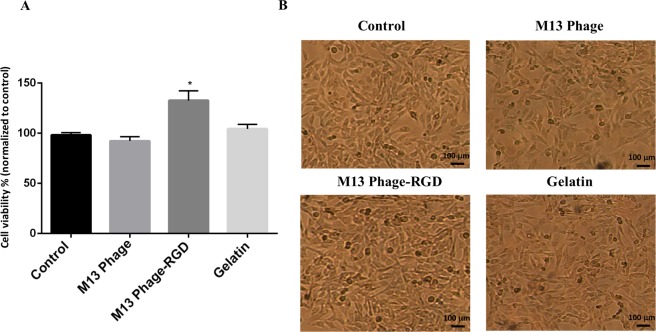
**(A)** Comparative analysis of survival rate of HUVECs after 48 hours culturing on coated (with M13 phage, M13 phage-RGD, and gelatin) and control surfaces by MTT assay. **(B)** Represents HUVECs morphology on coated (with M13 phage, M13 phage-RGD, and gelatin) and control surfaces with light microscope (40x). *Indicates that there is a significant difference between the experimental and control groups (*p* < 0.05).

HUVECs were seeded on coated with (M13 phage, M13 phage-RGD, and gelatin) and control wells in 96-well tissue culture plates at a density of 4 × 10^4^ cells.cm^−2^. After 48 hours culturing, the mitochondrial activity of HUVECs in each experimental group was evaluated by MTT assay. The MTT assay results showed that HUVECs in all experimental groups had a similar growth rate and viability pattern. However, the mitochondrial activity of HUVECs on M13 phage-RGD coated surface significantly increased in comparison with other groups (*p* < 0.05). But the number of HUVECs in M13 phage and gelatin groups was not significantly different from control group (Fig. 2A,B).

### Coated surfaces (M13 phage, M13 phage-RGD, and gelatin) have no cytotoxic effects on endothelial cells

**Figure 3:**
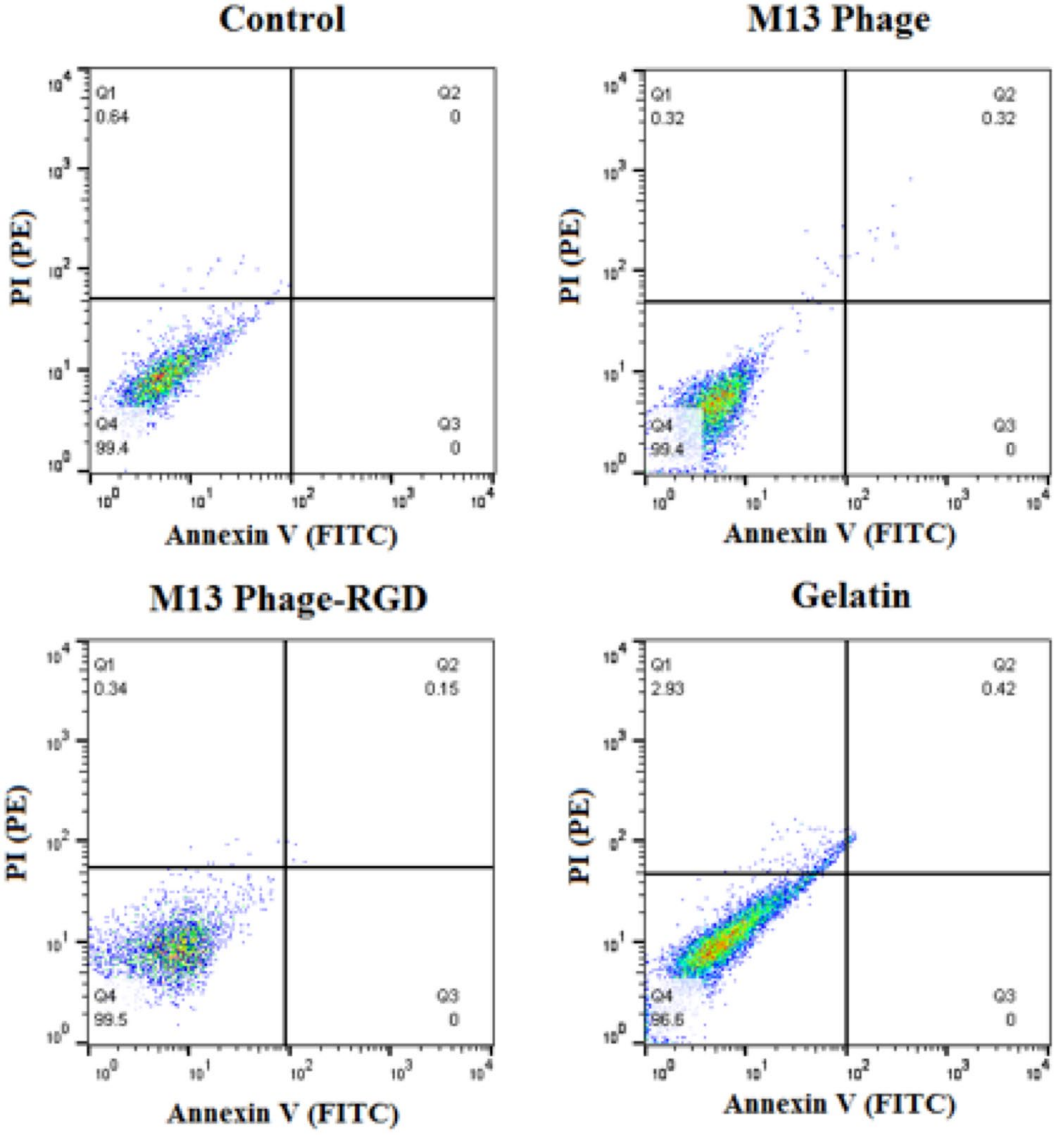
Flow cytometry analysis of HUVECs using Annexin V-FITC and PI-PE Dot plots, demonstrating the amount of necrosis and apoptosis of HUVECs after 48 hours culturing on coated (with M13 phage, M13 phage-RGD, and gelatin) and control surfaces. Q1: necrosis, Q2: late apoptosis, Q3: early apoptosis, and Q4: alive.

Annexin V-FITC/PI staining method was used to evaluate the apoptosis rate of HUVECs in each experimental group. Figure 3 represents dual parametric dot plots of stained cells, in which the lower left quadrant shows the viable cell population (negative for Annexin-V and PI), the lower right quadrant shows the population of apoptosis early stage (positive for Annexin-V and negative for PI), and the upper right quadrant demonstrates apoptotic cells or dead cell population (positive for Annexin-V and PI). According to the flow cytometry results shown in Fig. 3, selected surfaces in this study induced no apoptosis in endothelial cells after 48 hours culturing.

### The effects of phage, phage with RGD, and gelatin on HUVECs morphology

**Figure 4:**
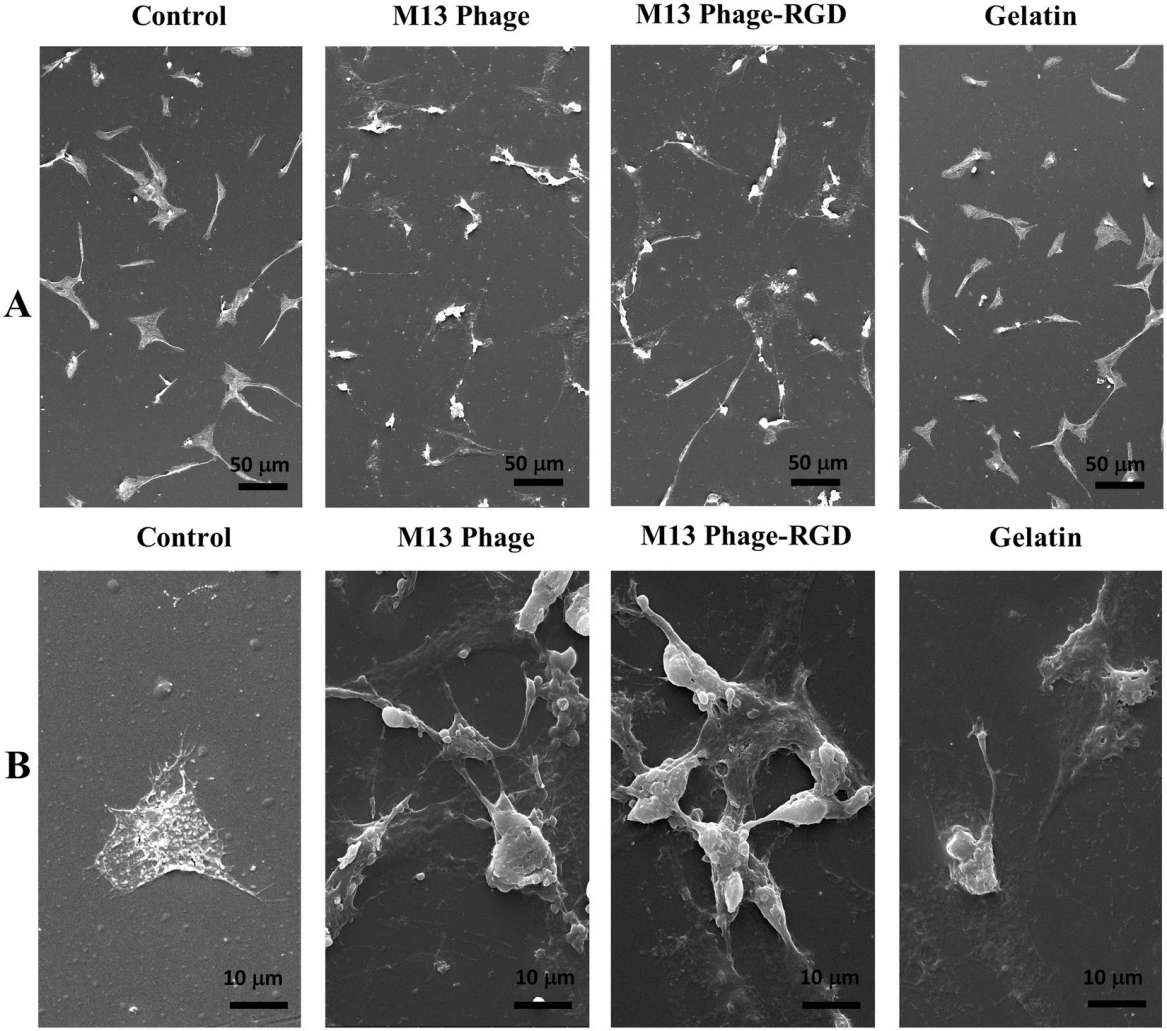
SEM micrographs of HUVECs after 48 hours culturing on coated (with M13 phage, RGD-M13phage, and gelatin) and control surfaces. significant difference is observed regarding the HUVECs morphology and size between the four different groups at two magnifications **(A)** 500x and **(B)** 3000x.

To determine how used surfaces in this study affect the HUVECs morphology, cells were grown on coated surfaces with M13 phage, M13 phage-RGD, and gelatin and unmodified control surface for 48 hours. The morphological changes in cells were investigated through scanning electron microscope after 48 hours culturing, as shown in Figure 4. The obtained results illustrated that HUVECs had the ability to adhere and spread on both M13 phage and M13 phage-RGD coated surfaces. Interestingly, the most significant morphological changes were observed in M13 phage-RGD group compared to gelatin and control groups. The results showed the pronounced protrusion of filopodia from HUVECs and increased attachment of cells to the underlying M13 phage and M13 phage-RGD coated surfaces. Importantly, the cells of M13 phage and M13 phage-RGD groups covered a vast area of the surfaces. In addition, SEM images showed that cells stretched filopodia toward each other in order to form a continuous cell layer.

### The effects of M13 phage, M13 phage-RGD, and gelatin on MMP-9 expression and activity

**Figure 5:**
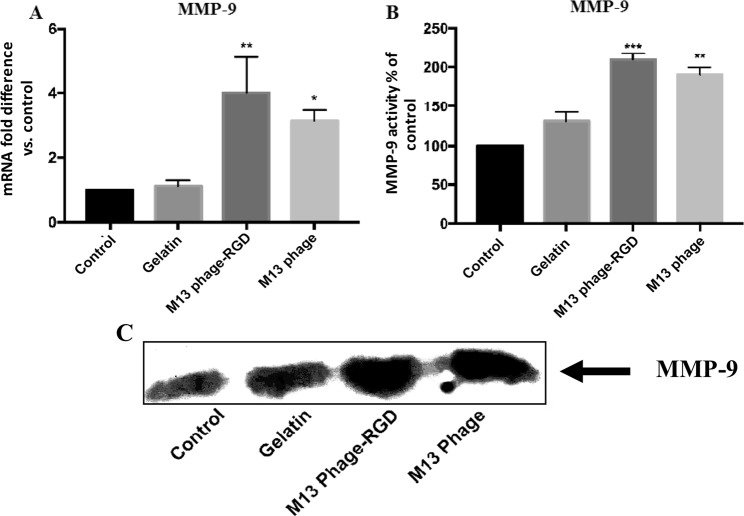
MMP9 gene expression and protein activity in HUVECs cultured on coated (with M13 phage, M13 phage-RGD, and gelatin) and unmodified control surfaces. **(A)** Represents a significant increase in MMP-9 gene expression by quantitative PCR in experimental group compared with control group. **(B)** Represents a schematic illustration of MMP-9 activity variations between the experimental groups. **(C)** In C, each band shows MMP-9 enzyme activity on gelatin by zymography assay. *p < 0.05, **p < 0.01, ***p < 0.001 demonstrate the presence of significant difference between the experimental and control groups.

One of the most important proteins during the process of angiogenesis is MMP-9 which has the ability to degenerate extracellular matrix. To determine whether MMP-9 gene expression and protein activity in HUVECs can be influenced by coated surfaces (M13 phage, M13 phage-RGD, and gelatin) and control unmodified surface, qRT-PCR and gelatin zymography assays were conducted. The qRT-PCR results showed that M13 phage-RGD group significantly stimulated the expression of MMP-9 in endothelial cells (Fig. 5A). To verify the results obtained from real-time PCR, gelatin-zymography analysis was conducted. The results showed that M13 phage-RGD and M13 phage coated surfaces significantly increased the potential gelatinolytic activity of MMP-9 in HUVECs culture supernatant (Fig. 5B,C).

### The effects of M13 phage, M13 phage-RGD, and gelatin on migratory ability of HUVECs

**Figure 6:**
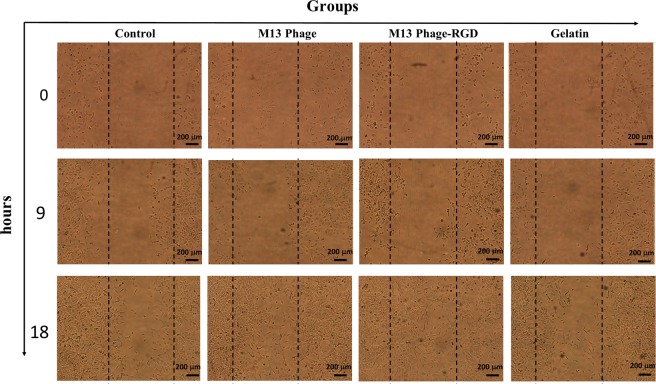
Microscopic analysis of HUVECs migration into the inter-space of scratched coated (with M13 phage, M13 phage-RGD, and gelatin) and control surfaces (40× magnification). According to figures, cell migration increased at 9 and 18 hours post scratch formation.

To further analyze the effect of selected surfaces on angiogenic potential of HUVECs, scratch-wound assay was used as an *in vitro* migration model. For excluding any possible effect of proliferation on migration, the effects of surfaces were assessed on migration of HUVECs prior to their doubling time (18 hours), and images were taken at 0, 9, and 18 hours after scratch gap creation on cell monolayers. The results pointed out that HUVECs cultured on M13 phage and M13 phage-RGD coated surfaces exhibited more dramatic increase in migration toward denuded area than cells seeded on gelatin and control groups. Furthermore, there was no visible difference between the cells seeded on gelatin coated and control surfaces (Fig. 6).

### The effects of M13 phage, M13 phage-RGD, and gelatin on VEGF-A and eNOS expression in HUVECs

**Figure 7:**
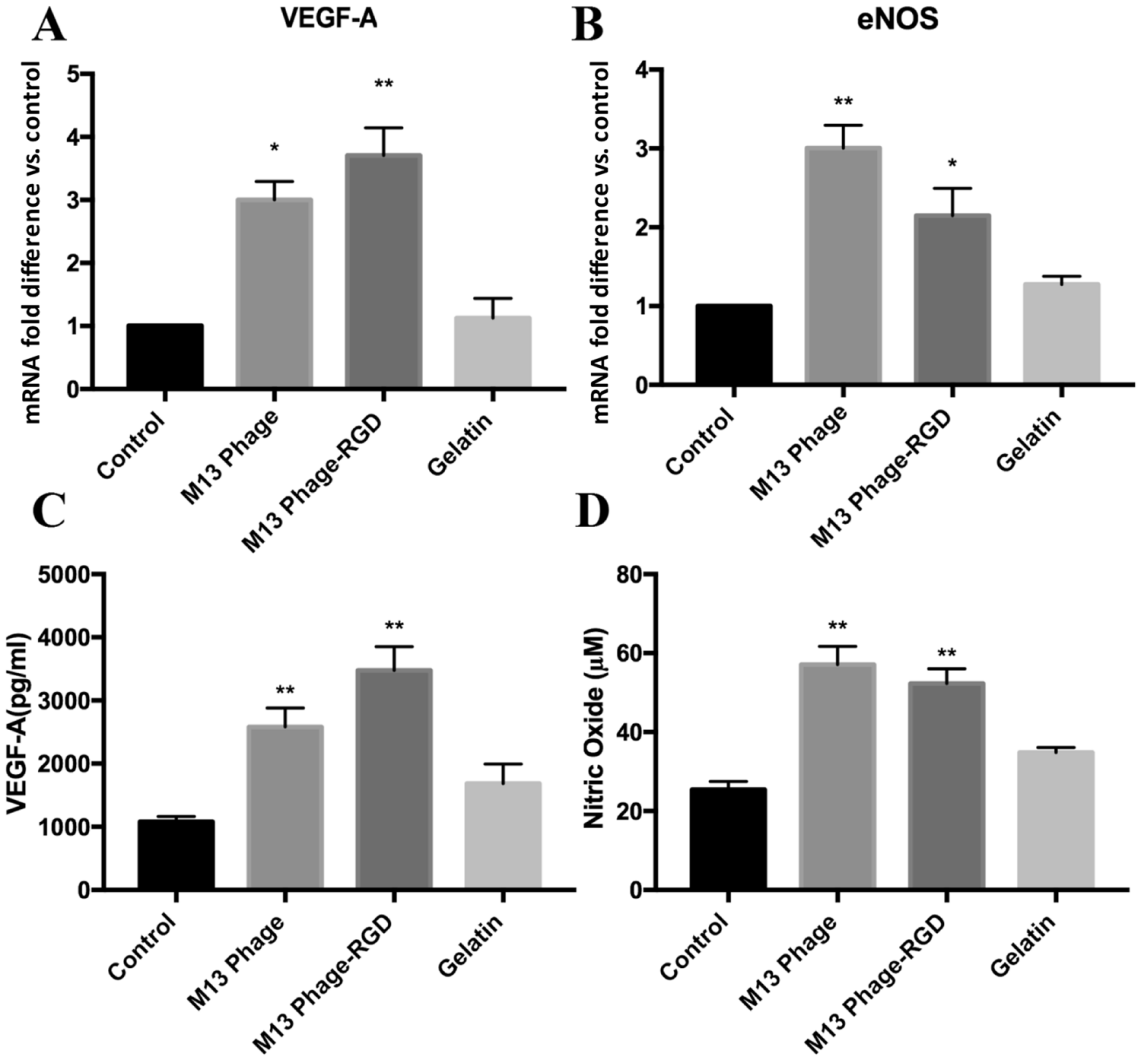
**(A,B)** Represent gene expression variations of VEGF and eNOS in HUVECs in response to coated (with M13 phage, M13 phage-RGD, and gelatin) and control surfaces. **(C,D)** Represent the changes in VEGF protein secretion and NO production in the supernatant of HUVECs in response to coated (with M13 phage, M13 phage-RGD, and gelatin) and control surfaces *p < 0.05, **p < 0.01, demonstrate the presence of significant difference between the experimental and control groups.

To further examine around the influence of coated surfaces (M13 phage, M13 phage-RGD, and gelatin) and control surface on the expression and secretion of angiogenic factors in HUVECs, the mRNA expressions of angiogenic-endogenic-associated genes, including VEGF-A and eNOS, were quantitatively evaluated by real-time PCR method. In addition, the levels of VEGF protein secretion and nitric oxide production were determined in culture supernatants of each experimental group by ELISA assay and nitric oxide assay, respectively. The results are depicted in Fig. 7.

It was found that the expression level of eNOS and VEGF-A transcripts was significantly increased in response to the cell culturing on M13 phage-RGD and M13 phage coated surfaces compared to control surface (*p* < 0.05), while there was no significant difference regarding eNOS and VEGF-A mRNA expression between the gelatin coated and control surfaces (Fig. 7A,B). To determine whether these changes were associated with the increased level of NO and VEGF-A protein production in the culture media, nitric oxide assay and ELISA test were performed. Regarding the VEGF-A protein secretion, it was found that after 48 hours culturing, the level of VEGF-A protein was significantly increased in the supernatant of cells cultured on M13 phage and M13 phage-RGD coated surfaces compared to those cultured on control surface (*p* < 0.05). Moreover, there were statistically significant differences regarding the production level of NO in the supernatant between the cells cultured on M13 phage and M13 phage-RGD coated surfaces and those cultured on control surface (*p* < 0.05). However, regarding the VEGF-A and NO secretion level, there was no statistically significant difference between the gelatin coated and control groups (Fig. 7C,D).

### The effects of M13 phage, M13 phage-RGD, and gelatin on the expression of VEGF receptors in HUVECs

**Figure 8:**
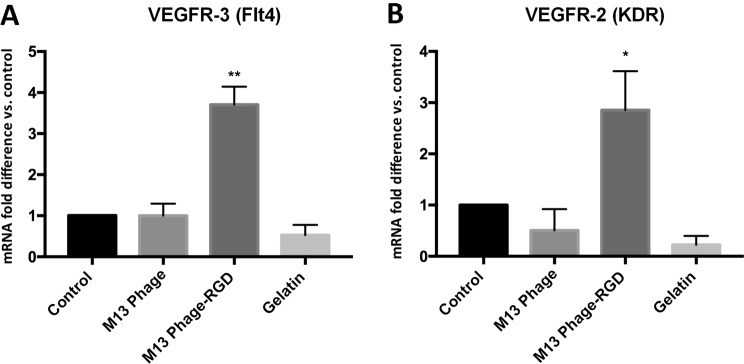
The analysis of VEGF receptors gene expression level in HUVECs in response to coated (with M13 phage, M13 phage-RGD, and gelatin) and control surfaces by real-time PCR. **(A)** Represents the expression levels of VEGFR-3 (Flt4) mRNA, and **(B)** represents the expression levels of VEGFR-2(KDR) mRNA in HUVECs. *p < 0.05, **p < 0.01, demonstrate the presence of significant difference between the experimental and control groups.

To investigate the mechanisms of different coatings associated with angiogenesis, VEGFR-2 and VEGFR-3 expression level in HUVECs was measured. The results of qRT-PCR demonstrated that the level of both VEGFR-2 and VEGFR-3 mRNAs expression was significantly upregulated in response to M13 phage-RGD coated surface in endothelial cells (Fig. 8A,B).

### The effects of M13 phage, M13 phage-RGD, and gelatin on intracellular ROS production in HUVECs

ROS is considered as another factor that can potentially induce angiogenesis in a microenvironment^20^. To further analyze the possible involvement of selected surfaces in HUVECs angiogenesis, cells were directly examined for the presence of intracellular ROS level using flow cytometry method.

**Figure 9:**
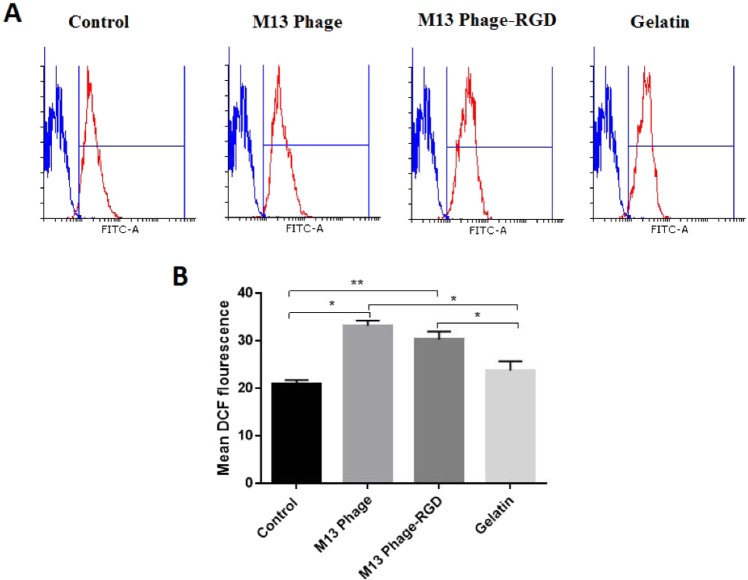
Flow cytometry analysis of intracellular ROS generation in HUVECs after 48 hours culturing on coated (with M13 phage, M13 phage-RGD, and gelatin) and control surfaces. (**A**) Histograms show the variation in intracellular ROS generation in HUVECs (**B**). Each bar demonstrates the mean ± SD of DCF fluorescent intensity in HUVECs. *p < 0.05, **p < 0.01, demonstrate the presence of significant difference between the experimental and control groups.

There were differences in the production level of generic ROS between the M13 phage, M13 phage-RGD, gelatin, and control groups (Fig. 9). The mean DCF fluorescent intensity of HUVECs after 48 h culture on control, M13phage, M13 phage-RGD and gelatin surfaces was 20.92, 33.14, 30.20 and 23.72, respectively. These results showed that the M13 phage and M13 phage-RGD surfaces could increase the level of intracellular ROS in HUVECs compared to gelatin and control surfaces.

### *In vivo* experiments

**Figure 10:**
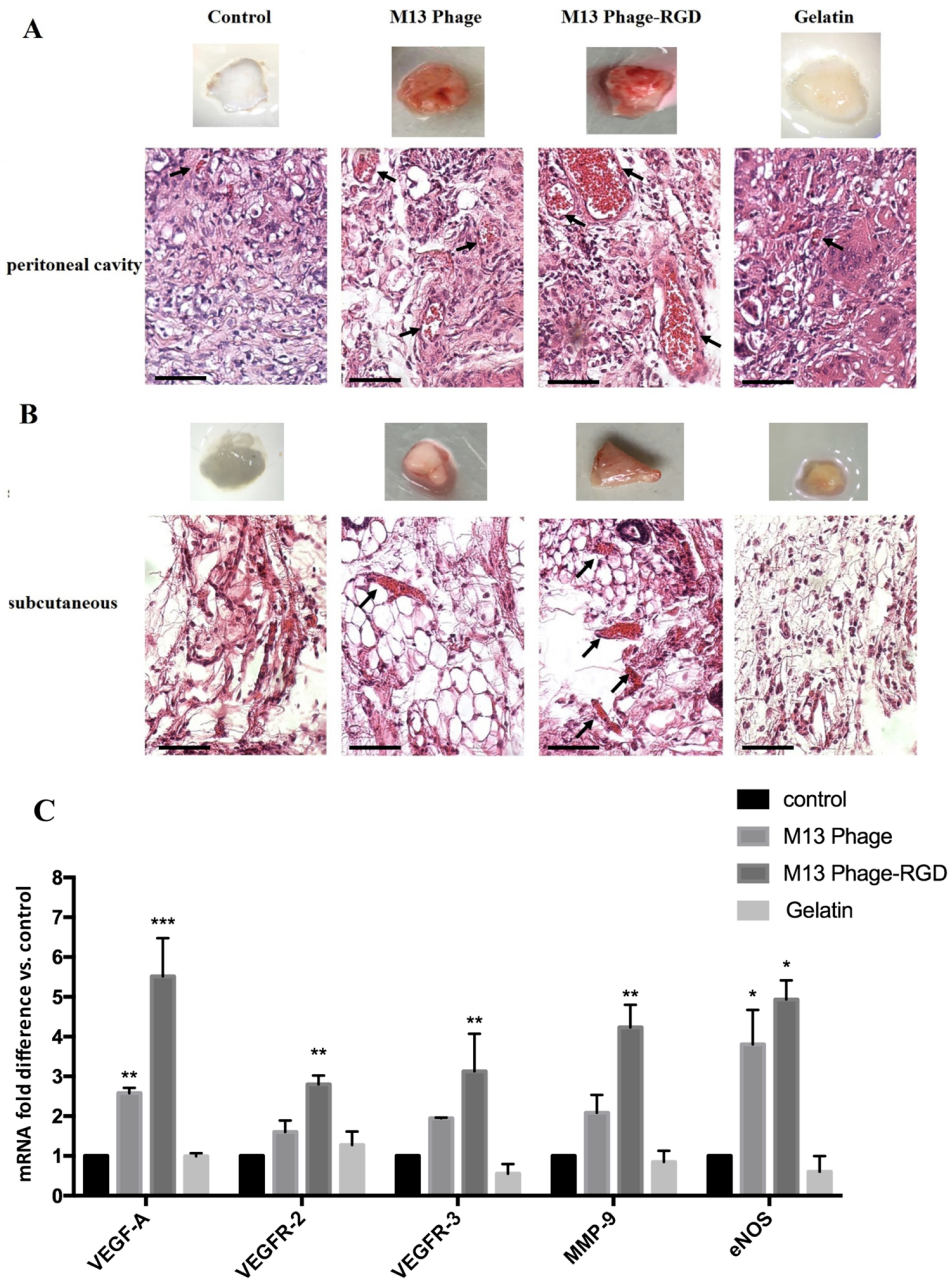
Macroscopic and histological analysis of PLGA scaffolds containing coated (with M13 phage, M13 phage-RGD, and gelatin) and control groups, implanted (**A**) in peritoneal cavity and (**B**) in subcutaneous after 10 days post implantation. Analysis of H & E staining shows the enhanced angiogenesis in implanted scaffolds containing M13 phage-RGD group compared to other groups. Arrows indicate the presence of tubular structures containing red blood cells in each experimental group (Scale bar demonstrate 100 μm/magnification 200x). (**C**) represents the expression level of VEGF-A, VEGFR-2, VEGFR-3, MMP-9, and eNOS genes measured by qPCR in implanted scaffolds containing coated (with M13 phage, M13 phage-RGD, and gelatin) and control groups after 10 days. *p < 0.05, **p < 0.01, ***p < 0.001 demonstrate the *p*resence of significant difference between the experimental and control groups.

Hematoxylin (H) and eosin (E) staining of samples showed an enhanced vascularization in the scaffolds of M13 phage-RGD group compared to other experimental groups. This event was observed with more obvious blood vessels containing red blood cells in tissue sections of M13 phage-RGD group scaffolds implanted in peritoneal cavity and subcutaneously (Fig. 10A,B).

Moreover, the qRT-PCR results indicated that the expression of angiogenesis related genes, including VEGF, VEGFR2, VEGFR-3, MMP-9, and eNOS, were significantly upregulated in the scaffolds of M13 phage-RGD group. It is worth nothing that the expression of VEGF and eNOS transcripts was significantly increased in the scaffolds of M13 phage group (Fig. 10C).

## Discussion

The current study clearly demonstrated that M13 phage-RGD could increase the angiogenic potential in *in vitro* and *in vivo* models. Based on the established potency of RGD peptide in mediating multiple activities in cell, including cell adhesion, proliferation, and differentiation, it has been widely used in designing smart biomaterials for tissue engineering^21^. M13 phage possess unique characteristics compared to other classes of nanofibers because as a building block, it could be used for creating special nanostructures through self-assembly^22^. Phage-based 2D and 3D scaffolds was previously reported to display good biocompatibility in the fibroblast cell line model. These scaffolds represent chemical and physical characteristics required for influencing cells growth patterns^23^. Based on the obtained results, it was clear that M13 phage and RGD peptide are involved in angiogenesis induction. In addition, it was showed that M13 phage in combination with RGD peptide could induce angiogenesis more than M13 phage and gelatin.

*In vitro* results of the current study demonstrated that M13 phage and RGD peptide could modulate the morphology and shape of the cells. In addition, M13 phage-RGD coated surface influenced cell proliferation much more than the wild-type M13 phage and gelatin coated and control surfaces. Accordingly, it can be hypothesized that M13 phage in combination with RGD motif is able to strongly induce the angiogenesis through the proliferation of endothelial cells. It is clear that endothelial cells proliferation is the key process in neoangiogenesis (formation of new blood vessels)^24^. In addition, the ability of M13 phage-RGD coated surface to induce adhesive changes in cells could modulate the important angiogenesis related behaviors in endothelial cells. Hence, in the current study, a mixture of RGD peptide and M13 phages was used to form an ECM for angiogenesis induction.

In this study, the effects of selected surfaces on the endothelial cells apoptosis were also explored. According to the results, Annexin V-FITC/PI staining confirmed that the M13 phage and M13 phage-RGD coated surfaces were not toxic for HUVECs at the tested concentrations.

Remarkably, the obtained results demonstrated that M13 phage-RGD coated surface was able to promote better cell adhesion and spreading than the other surfaces. RGD peptide motif displayed on most of common extracellular matrix proteins including laminin, fibronectin, vitronectin, von Willebrand factor, osteopontin and etc. The cell surface attachment proteins, such as integrins, bind to the RGD motif within ECM glycoproteins and lead to the increased attachment of cells to their surrounding ECM^25^. The M13 phage-RGD coated surface had the advantage of providing essential anchoring cues for cells and thereby contributing to the promotion of cell-cell and cell-matrix adhesions^21^.

This led to the formation of a greater cell-cell interactions and a higher cellular attachment on M13 phage-RGD coated surface. In contrast, the gelatin coated and unmodified control surfaces displayed lower surface area and therefore were not able to make available adequate attachment sites for the cells. It has been reported that the endothelial cells strong attachment capability to ECM proteins through increased adhesion to the RGD sequence^26^. According to the present study results, the elevated adhesion of HUVECs on M13 phage-RGD coated surface can be attributed to the fact that RGD peptide and M13 phage might have achieved endothelial-specific activity by interacting with endothelial cells.

The involvement of reactive oxygen species (ROS) (such as superoxide anion and hydrogen peroxide) in angiogenesis process and VEGF expression has been evidenced by many research groups^27^. In HUVECs, increased accumulation of ROS has been reported as an important factor for the promotion of angiogenesis^28^. Previous report also indicated that ROS affects VEGF-stimulated activation of VEGF Receptor 2 through its dimerization and autophosphorylation^28^. It is interesting to mention that VEGF induces migration and proliferation of endothelial cells by increasing the levels of intracellular ROS^29–32^. Other results support the involvement of ROS, produced by endothelial cells, in the expression of VEGF and VEGF Receptor (VEGFR) 2 in tumor microenvironment^33^. This study finding indicated that the production of intracellular ROS and the expression of VEGFR-2 were also elevated upon the cells exposure to M13 phage-RGD coated surface. These results are consistent with the previous reports regarding the angiogenesis promoting role of ROS and VEGFR-2 and clearly demonstrated the effective involvement of M13 phage and RGD peptide in HUVECs functions.

In the current study, the increased expression level of VEGF-A at the mRNA and protein level was shown in M13 phage-RGD and M13 phage groups. VEGFs have been well known for their pro-angiogenic potency through the stimulation of endothelial cell proliferation, migration, and tube formation activities^34,35^. In this study, increased levels of eNOS mRNA was also observed in M13 phage-RGD group. eNOS gene expresses constitutively in endothelium and plays a critical role in regulating the growth pattern of endothelial cells through its contribution to the synthesis of basal NO^36^.

Interestingly, there is a bidirectional relationship between the expression of angiogenic factors and NO, in which angiogenic factors such as VEGF have a role in elevating the expression of eNOS and subsequently production of NO in endothelium to increase angiogenesis^37^. Furthermore, nitric oxide (NO) is considered as a main regulator for endothelial cell growth, and previous reports showed that NO induces the expression of VEGF in endothelial cells^38^.

The obtained results pointed out that the expression levels of angiogenesis-associated genes (including VEGF, VEGF-R2, VEGF-R3 and eNOS) were strongly increased in HUVECs cultured on M13 phage-RGD coated surface compared to other groups, indicating a better angiogenic potential of M13 phage-RGD than the other groups. Additionally, earlier reports showed that VEGF upregulates the expression of integrin αvβ3 by endothelial cells. This type of integrin binds to the proteins containing Arg-Gly-Asp (RGD) motif in their structures, and it allows the migration and adhesion of endothelial cells^39^. Thus, it can be concluded that the nanostructures of M13 phage-RGD coated surface are able to prepare more favorable local microenvironment for HUVECs to promote blood vessel formation.

Indeed, angiogenesis process in a tissue initiates by the proliferation, extracellular matrix degradation, and migration of endothelial cells^40^. Matrix metalloproteinases (MMPs) are a class of proteins breaking down the extracellular matrix proteins. Interestingly, VEGF signaling through VEGF Receptor 2 leads to the expression of endothelial cell-derived MMPs, especially MMP9^41^. In the current study, it was demonstrated that M13 phage-RGD mediates MMP9 activity in endothelial cells. These results confirm that M13 phage-RGD coated surface induces angiogenesis in endothelial cells via the induction of VEGF and MMPs expression.

According to *in vitro* results, *in vivo* results demonstrated that the expression of VEGF-A, VEGFR-2, VEGFR-3, MMP9, and eNOS genes were significantly upregulated in M13 phage-RGD group PLGA/PCL scaffolds. It is worth nothing that the histological observations of H & E analysis showed more obvious blood vessels formation in the implanted PLGA/PCL scaffold of M13 phage-RGD group. This observation suggests that M13 phage along with RGD peptide is able to promote angiogenesis *in vivo* and could be served as a biomaterial in designing tissue engineering scaffolds.

## Conclusion

**Figure 11:**
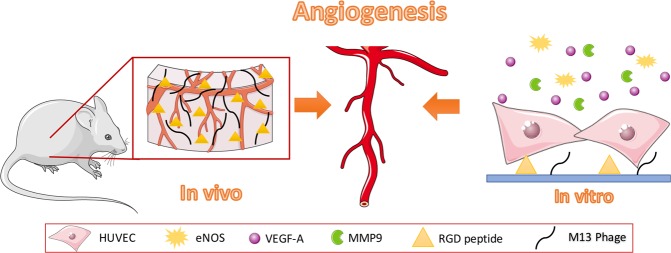
M13 phage along with RGD peptide is able to provide a microenvironment for promoting angiogenesis both *in vitro* and *in vivo*.

In conclusion, the function of endothelial cells significantly changed with the exposure to M13 phage-RGD and M13 phage coated surfaces more efficiently than the exposure to gelatin coated surface. The results pointed out that M13 phage-RGD induced the migratory potentials of endothelial cells by increasing the attachment of HUVECs. It was also demonstrated that HUVECs displayed a good spreading morphology, enhanced cell-cell communication, and cell-substrate adhesion on M13 phage-RGD group. Elevated levels of VEGF, MMP9, NO, and VEGF receptors in M13 phage-RGD group led to the increased angiogenic potential of HUVECs. *In vivo* results of this study showed more angiogenic potential in the M13 phage-RGD scaffolds than the other groups. To sum up, this study results may be used as a guide for the development of new materials with bacteriophage scaffolds aiming to enhance angiogenic potential of cells for tissue regeneration (Fig. 11).

## Supplementary information

Supplementary Information
