# Supplementary material for: Promotion of angiogenesis by M13 phage and RGD peptide in vitro and in vivo
Source: Sci Rep. 2019 Aug 1;9:11182. doi: 10.1038/s41598-019-47413-z (PMC6672002; doi:10.1038/s41598-019-47413-z)

Promotion of angiogenesis by M13 phage and RGD peptide in vitro and in vivo

Zohreh Safari[1], Sara Soudi*[2], Nazli Jafarzadeh[1], Ahmad Zavaran Hosseini[2], Elham Vojoudi[3], Majid Sadeghizadeh[1]*

1-Department of genetics, Faculty of Biological Sciences, Tarbiat Modares University, Tehran, Iran

2-Department of Immunology, Faculty of Medical Sciences, Tarbiat Modares University, Tehran, Iran

3-Department of Regenerative Medicine, School of Advanced Technologies in Medicine, Tehran University of Medical Sciences, Tehran, Iran

Supplementary Table 1: Target genes and primer sequences for qRT-PCR.

Target genes	Forward 5' to 3'	Revers 3' to 5'
Human VEGF-A	AACGTACTTGCAGATGTGACAAG	GCTCGGTGATTTAGCAGCAAGAA
Human VEGFR-2	CCAGTCAGAGACCCACGTTT	TCCAGAATCCTCTTCCATGC
Human VEGFR-3	CTGGTAGGGGAGAAGCTGGTC	GGACAGTGCCTTTCCATCCTT
Human MMP-9	GCCCCAGCGAGAGACTCTAC	CTGGTACAGGTCGAGTACTCC
Human eNOS	CCTTCGCTCGGCCATCACA	CCAGGGCTGCAAACCACTC
Human GAPDH	CCGAGCCACATCGCACAG	GGCAACAATATCCACTTTACCAG
Mouse VEGF-A	GGAGATCCTTCGAGGAGCACTT	GGCGATTTAGCAGCAGATATAAGAA
Mouse VEGFR-2	CTGGTTGTGAATGTCCCACC	CTTGTTTGGCCGGGTCTGTAG
Mouse VEGFR-3	GAGGCCAACAATGGGATTCAG	TTGTACCATTGGAACTCCGGT
Mouse MMP-9	GCGTGTCTGGAGATTCGACT	CTTGGTACTGGAAGATGTCGT
Mouse eNOS	ATGGAGCATCCCAAGTACGAG	CTCCAGGATGTTGTAGCGCTG
Mouse GAPDH	CCTGGAGAAACCTGCCAAGTA	GGCATCGAAGGTGGAAGAGT

Gel figure:

The unmodified figure of the gel and the cropped figure (used in manuscript) are provided as you requested.

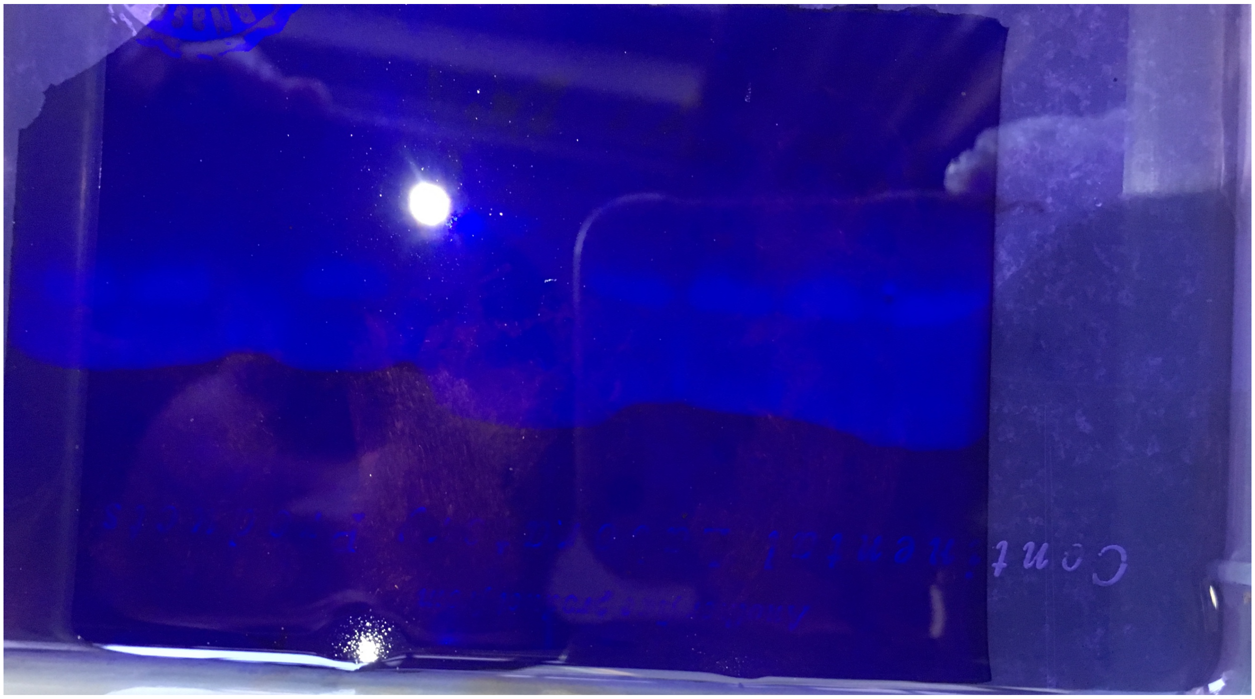

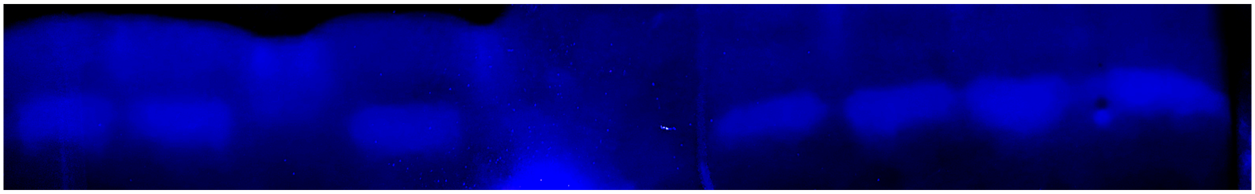

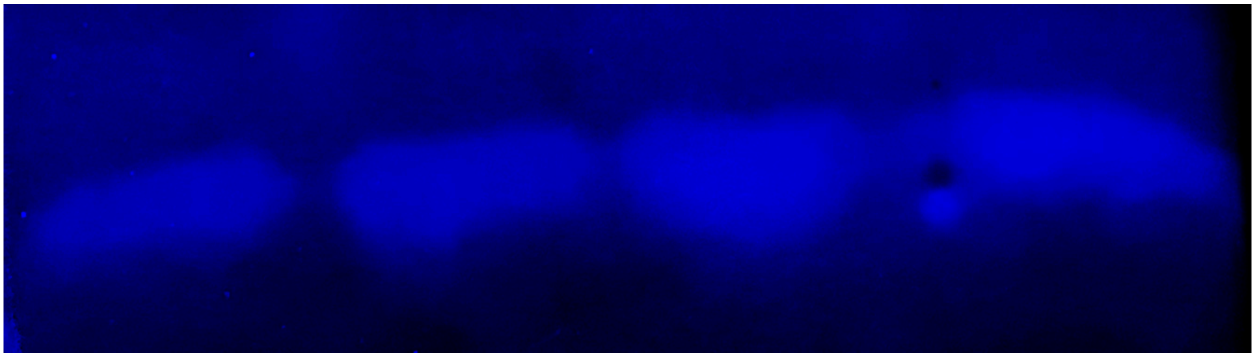

Supplement: Supplementary file 1 — Supplementary Information [file 41598_2019_47413_MOESM1_ESM.pdf]
